# Novel dye-assisted localization of the minor papilla via a percutaneous drainage catheter for pancreatic duct disruption

**DOI:** 10.1055/a-2897-7647

**Published:** 2026-06-30

**Authors:** Toji Murabayashi, Mayu Kawabata, Shinya Sugimoto

**Affiliations:** 1 Department of Gastroenterology 37071 Ise Red Cross HospitalIseJapan

## Case description



**Video 1** Dye-assisted localization of the minor papilla through a percutaneous drainage catheter, followed by endoscopic pancreatic duct drainage via the minor papilla.


Endoscopic pancreatic duct drainage via the minor papilla is occasionally required when drainage via the major papilla is difficult or anatomically unsuitable. However, cannulation of the minor papilla is often technically difficult because it is endoscopically inconspicuous. Several dye-assisted techniques have been reported for localization of the minor papilla, including topical dye spraying, intraductal dye injection, and EUS-guided methylene blue pancreatography. 1​ 2​ ​3​ We herein report a case of traumatic complete disruption of the Wirsung duct in which injection of indigo carmine-containing contrast medium through a percutaneous retroperitoneal drainage catheter enabled endoscopic identification of the minor papilla and successful pancreatic duct drainage via the minor papilla.

**Fig. 1:**
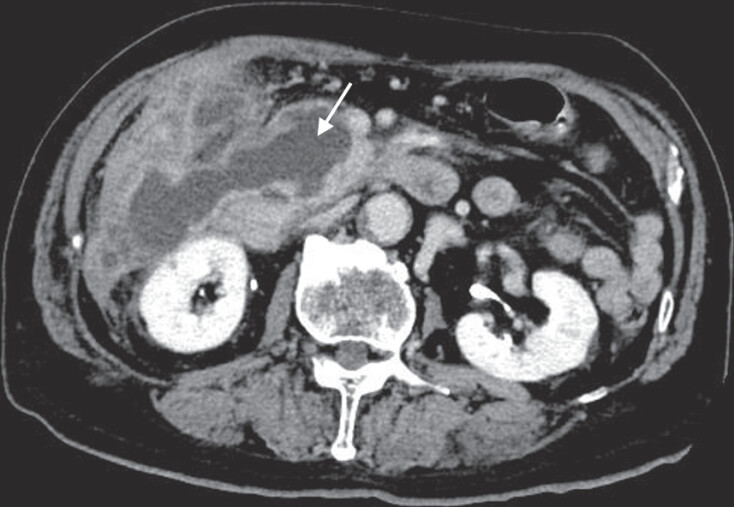
Contrast-enhanced computed tomography showing a large retroperitoneal fluid collection adjacent to the pancreatic head (arrow).

**Fig. 2:**
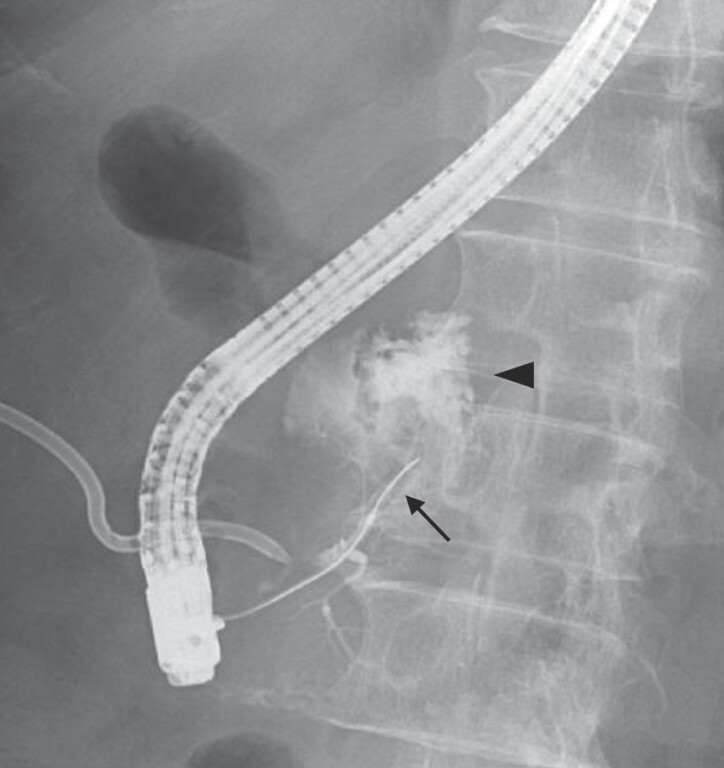
Fluoroscopic image during endoscopic retrograde pancreatography via the major papilla showing complete disruption of the Wirsung duct (arrow), with contrast leakage into the retroperitoneal cavity (arrowhead) and no opacification of the upstream pancreatic duct.

**Fig. 3:**
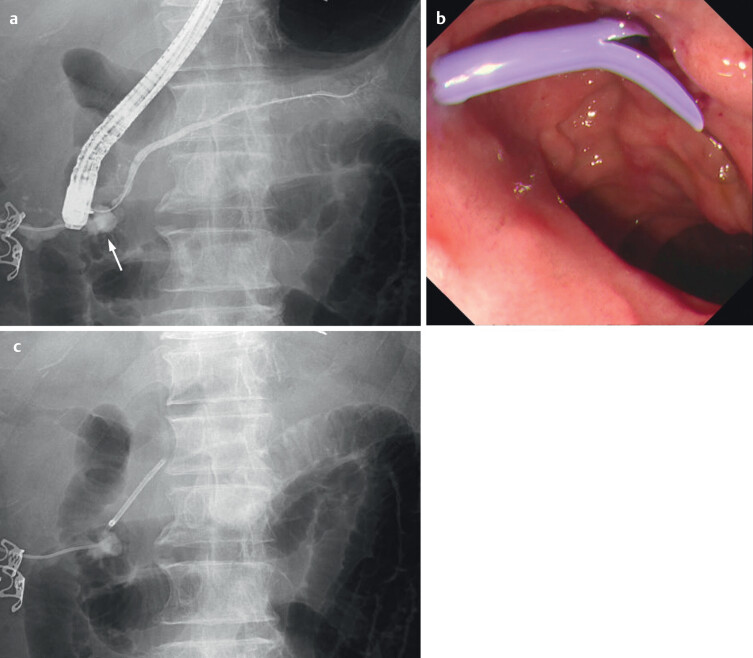
Endoscopic pancreatic duct drainage via the minor papilla using dye-assisted localization. ( **a** ) Fluoroscopic image during pancreatography via the minor papilla showing opacification of the Santorini duct and the upstream pancreatic duct, with contrast leakage into the retroperitoneal cavity (arrow) and no flow into the Wirsung duct. ( **b** ) Endoscopic image showing a plastic stent placed via the minor papilla. ( **c** ) Fluoroscopic image confirming plastic stent placement via the minor papilla.

A 77-year-old man was referred to our hospital for management of traumatic pancreatic duct injury. Computed tomography showed a large retroperitoneal fluid collection adjacent to the pancreatic head ( Fig. 1 ). Percutaneous drainage of the collection was performed, and the amylase level in the drained fluid was 80,900 U/L, confirming pancreatic juice leakage. Endoscopic retrograde pancreatography via the major papilla showed complete disruption of the Wirsung duct, with contrast leakage into the retroperitoneal cavity and failure of guidewire passage across the disruption site ( Fig. 2 ). Several days later, pancreatic duct drainage via the minor papilla was performed ( Fig. 3 ; Video 1 ). Because the minor papilla was endoscopically inconspicuous, contrast medium mixed with indigo carmine was injected through the percutaneous retroperitoneal drainage catheter. The dye emerged from the minor papilla, allowing direct endoscopic localization. A guidewire was then advanced through the minor papilla deep into the upstream pancreatic duct, and a 7-Fr plastic stent was placed across the Santorini duct via the minor papilla. Pancreatic juice leakage improved, allowing removal of the percutaneous drainage catheter 2 weeks later. The pancreatic stent was removed 4 months later, with no subsequent recurrence of pancreatic juice leakage.

Endoscopy_UCTN_Code_TTT_1AR_2AK
